# Chronic inflammatory activity in women with normogonadotropic anovulation complicated by subclinical thyroid dysfunction: a prospective cohort study

**DOI:** 10.3389/fendo.2026.1772362

**Published:** 2026-03-09

**Authors:** Rafal Baran, Justyna Brodowicz, Krzysztof Skotniczny, Robert Jach, Iwona Gawron

**Affiliations:** 1 University Hospital in Krakow, Clinical Department of Gynecological Endocrinology and Gynecological Oncology, Krakow, Poland; 2 Jagiellonian University Medical College, Faculty of Medicine, Chair of Clinical Biochemistry, Krakow, Poland; 3 Jagiellonian University Medical College, Faculty of Medicine, Chair of Gynecology and Obstetrics, Krakow, Poland

**Keywords:** anti-müllerian hormone, chronic inflammation, normogonadotropic anovulation, polycystic ovary syndrome, subclinical hypothyroidism, thyroid autoimmunity

## Abstract

**Purpose:**

To investigate the impact of subclinical hypothyroidism (SCH) and thyroid autoimmunity (TAI) on systemic inflammatory activity in women with normogonadotropic anovulation, comparing polycystic ovary syndrome (PCOS) and hypothalamic-pituitary-ovarian dysfunction (HPOD), and to examine the correlations of inflammatory parameters with thyroid, metabolic, and ovarian indices.

**Methods:**

Concentrations of C-reactive protein (CRP), tumor necrosis factor-α (TNF-α), interleukin-6, interleukin-1β, and interleukin-10 were prospectively measured in anovulatory women and compared between those with PCOS and HPOD, considering the influence of SCH and TAI. Multiple regression analysis was performed to evaluate the relationships among thyroid dysfunction, inflammatory parameters, and indices of both metabolic and ovarian function.

**Results:**

Both SCH and TAI independently increased TNF-α concentrations across the entire cohort (p=0.005 and p=0.018) and within the PCOS arm (p=0.018 and p=0.039). TAI significantly elevated the IL-1β/IL-10 ratio in the entire cohort (p=0.009) and in PCOS arm (p=0.005), with significant interaction effects between SCH and TAI in both groups (p=0.026 and p=0.017). No significant associations were found in the HPOD arm. In the overall cohort, CRP concentrations, which positively correlated with BMI, insulin resistance indicators, and dyslipidemia, and negatively with estradiol, were significantly higher in SCH (p-values <0.001). In PCOS, interleukin-1β and TNF-α levels, positively correlated with some insulin resistance indicators and TNF-α with AMH, were also significantly elevated in both SCH and TAI.

**Conclusions:**

SCH and TAI independently and synergistically promoted chronic inflammation in normogonadotropic anovulation, particularly in PCOS, through elevated TNF-α and disrupted interleukin-1β/IL-10 balance, with significant implications for metabolic health and ovarian function.

## 1 Introduction

Chronic low-grade inflammation has been increasingly recognized as an inherent feature of endocrine disorders affecting reproductive health, potentially linking the intricate hormonal and metabolic pathways involved in their development (1, 2). This systemic inflammation negatively impacts the prognosis, leading to increased morbidity and mortality (3). Recent scientific attention has focused on inflammation associated with polycystic ovary syndrome (PCOS), a complex endocrine disorder affecting 10–13% of women of reproductive age (4). PCOS is characterized by ovulatory dysfunction, hyperandrogenism, and polycystic ovarian morphology (PCOM) (4). Furthermore, this condition has been linked to elevated serum inflammatory markers, including C-reactive protein (CRP), interleukin-6 (IL-6), and tumor necrosis factor-alpha (TNF-α) (5, 6). Although the etiology of PCOS is still under investigation, excess androgens and insulin resistance have been recognized as major pathogenic factors (7, 8), likely contributing to the pathophysiology of concomitant inflammation (9). This inflammation likely drives metabolic disorders and ovarian dysfunction, triggering interrelated pathological processes that ultimately increase the long-term health risks associated with anovulatory infertility, metabolic syndrome, and endometrial cancer (10–15). Hypothalamic-pituitary-ovarian dysfunction (HPOD) represents another normogonadotropic anovulatory condition (4), yet the associated inflammatory profile and its impact on ovulation and metabolic status have not been thoroughly researched. Metabolic and hormonal functions may be influenced by thyroid activity. While overt hypothyroidism justifies the administration of L-thyroxine to enhance fertility and metabolic parameters (16), the efficacy of treating subclinical thyroid dysfunction to improve gynecological and obstetric outcomes or prevent metabolic syndrome remains unsubstantiated by scientific evidence (17). The issue is noteworthy due to the increased prevalence of subclinical hypothyroidism (SCH) and thyroid autoimmunity (TAI) among reproductive-age women with ovulatory disorders, with rates rising from 2.4% to 46% for SCH and from 7.5% to 26.9% for TAI (18–20). The relationship between thyroid function and metabolic status appears bidirectional. Obesity and insulin resistance may both contribute to and be influenced by mild thyroid hormone deficiency, potentially through inflammatory mechanisms (21). Additionally, autoimmune-related inflammation may play a role in the co-occurrence of TAI with these metabolic disorders, as the molecular mechanisms underlying these associations remain largely unexplored (21, 22). The pathophysiology of inflammation in the context of TAI and SCH, along with their combined effects on metabolic and ovarian parameters, has not yet been thoroughly investigated (23). No recommendations have been developed thus far for the use of inflammatory marker concentrations to inform diagnosis, treatment, or prevention of complications related to these conditions.

Demonstrating the potential impact of inflammation in thyroid dysfunctions on metabolic and ovarian parameters in women with ovulatory disorders may have important clinical implications. Improved understanding of these biological interactions could contribute to increased ovulation rates, better obstetric outcomes, and the alleviation of metabolic complications through behavioral and pharmacological interventions (24).

The objective of the study was to measure and compare concentrations of selected inflammatory parameters, specifically CRP, TNF-α, IL-6, interleukin-1β (IL-1β) and interleukin-10 (IL-10), in women with normogonadotropic anovulation. The study aimed to distinguish between PCOS and HPOD, while also taking into account the concurrent presence of SCH and TAI. Additionally, the associations between inflammatory markers and metabolic and ovarian parameters in these conditions were assessed.

## 2 Materials and methods

The single-center prospective cohort study was conducted among women with menstrual irregularities between February and September 2024. The study was approved by the Bioethics Committee of Jagiellonian University (no. 1072.6120.292.2022) and conducted in accordance with the Declaration of Helsinki. Informed written consent was obtained from all participants. The study was recorded in the Protocol Registration and Results System on ClinicalTrials.gov (NCT05842096). The following inclusion criteria were applied: i) age between 18 and 45 years, ii) cycle length of < 21 days or > than 35 days. Prolonged intermenstrual intervals were deemed indicators of anovulation, while shorter intervals indicated ovulatory dysfunction, which was also recognized as a criterion for study inclusion. The exclusion criteria were as follows: i) absence of at least one ovary, ii) a previously diagnosed and treated thyroid or autoimmune disorders. The comprehensive diagnostic evaluation included a medical interview, physical examination with gynecological speculum and bimanual assessments, and extensive biochemical blood analysis. Based on the medical history, it was confirmed that none of the participants used anti-inflammatory drugs to treat inflammatory conditions. Ultrasonographic evaluation of reproductive organs was performed with a Samsung WS80A system (Samsung Electronics, Suwon, Korea) equipped with volume endocavity (EV2-10A) and convex (CV1-8A) transducers.

PCOS diagnosis followed the Rotterdam criteria (8), and participants were categorized into phenotypes based on the following characteristics: A - irregular menstruation, hyperandrogenism, defined as elevated serum testosterone concentration or free androgen index (FAI) > 5, and PCOM; B - irregular menstruation and hyperandrogenism, and D - irregular menstruation and PCOM. The absence of women with phenotype C resulted from irregular menstruation being an inclusion criterion. The presence of at least 20 follicles measuring 2 to 9 mm in diameter and/or an ovarian volume of at least 10 ml, in the absence of a corpus luteum, dominant follicle, or functional cyst, indicated PCOM (8). FAI was calculated to determine bioavailable testosterone by dividing the total testosterone concentration (nmol/l) by the SHBG concentration (nmol/l). Normogonadotropic anovulation that did not meet the diagnostic criteria for PCOS was classified as HPOD (4).

In order to analyze the influence of thyroid dysfunction on systemic inflammatory activity, as measured by the concentrations of inflammatory parameters, SCH and TAI were defined. SCH was characterized as a TSH concentration exceeding 2.5 mIU/ml (25) with normal free thyroid hormone concentrations, whereas TAI was identified by the presence of circulating thyroid autoantibodies (ATA): thyroid peroxidase antibodies (TPOAb) and/or thyroglobulin antibodies (TGAb). TPOAb positivity was established as concentrations above 34 IU/ml and TGAb positivity as concentrations exceeding 115 IU/ml. The reference range for fT3 was 2.5–10 pmol/L and for fT4 10–26 pmol/L.

The homeostasis model assessment for insulin resistance (HOMA-IR) was determined by multiplying FI (µIU/ml) by FG (mmol/l) and dividing by 22.5 (26).

Women with suspected hyper- or hypogonadotropic hypogonadism, hyperprolactinemia, virilizing tumors, or other adrenal disorders were excluded from analysis.

### 2.1 Determination of the concentrations of biochemical parameters

Biochemical tests were conducted on a 10 ml venous blood sample collected after at least eight hours of fasting using the Cobas PRO/e801 analyzer (Roche Diagnostics, Basel, Switzerland). The serum concentrations of follicle-stimulating hormone (FSH), luteinizing hormone (LH), prolactin (PRL), Anti-Müllerian hormone (AMH), estradiol, testosterone, sex hormone binding globulin (SHBG), dehydroepiandrosterone sulphate (DHEA-S), vitamin D, fasting (FI) and at 120 minutes of the 75 g glucose tolerance test (120’75OGTTI) insulin, thyroid-stimulating hormone (TSH), free triiodothyronine (fT3), free thyroxine (fT4), TPOAb and TGAb were assessed by applying the electrochemiluminescence immunoassay (ECLIA). Fasting glucose (FG) concentration and at 120 minutes of the 75 g glucose tolerance test (120’75OGTTG), as well as triglycerides (TG) and total cholesterol (TC) concentrations were measured with an enzymatic method. High-density lipoprotein-cholesterol (HDL), alanine (ALT) and aspartate (AST) transaminases concentrations were evaluated by means of a spectrophotometric method. CRP concentration was determined using an immunoturbidimetric method. Low-density lipoprotein cholesterol was estimated using the Friedewald equation, acknowledging its reduced accuracy at higher triglyceride concentrations and lower LDL-C measurements.

### 2.2 Determination of the concentrations of cytokines

Blood samples collected in anticoagulant-free vacuum tubes (S-Monovette, Sarstedt AG) were left to coagulate for 30 minutes at room temperature. Following centrifugation at 3000 revolutions per minute for 15 minutes, the serum supernatant was aspirated, transferred into Eppendorf Safe-Lock Tubes (Eppendorf, Hamburg, Germany) in 300 µl aliquots, and stored at -20 °C until analysis. The serum concentrations of IL-1β, IL-10, and TNF-α were quantified using enzyme-linked immunosorbent assay (ELISA) kits (Quantikine ELISA Kits, R&D Systems, Bio-Techne, Minnesota, USA) according to the manufacturer’s instructions. The assay sensitivities were 1.0 pg/ml for IL-1β, 3.9 pg/ml for IL-10, and 4.0 pg/ml for TNF-α. The intra-assay and inter-assay coefficients of variation (CV%) were 4.75% and 5.6% for IL-1β, 3.7% and 6.9% for IL-10, and 2.6% and 7.7% for TNF-α, respectively. Absorbance readings were obtained at 450 nm, with a reference wavelength of 565 nm, using a BioTek 800 TS microplate reader (BioTek Instruments, Winooski, Vermont, USA), with data collected via BioTek Gen5 software. Standard curves were generated using a four-parameter logistic regression model. The concentration of IL-6 was determined using an automated Roche Cobas PRO/e801 analyzer with ECLIA (Elecsys IL-6, Roche Diagnostics, Basel, Switzerland). The sensitivity of the IL-6 assay was 1.5 pg/ml, and the intra- and inter-assay CV% values were 2.54% and 2.02%, respectively. To minimize bias, the laboratory analyst was blinded to the participants’ diagnoses.

### 2.3 Study design and groups

To evaluate how thyroid function impacts inflammatory parameters in women with normogonadotropic anovulation, the cohort was divided into two study arms: i) PCOS with its phenotypes and ii) HPOD. Within each arm, subpopulations with i) SCH and ii) TAI were identified. The study analyzed the effects of these thyroid dysfunctions on inflammatory, metabolic, and ovarian parameters across the entire cohort as well as within the PCOS and HPOD arms, thereby enabling a detailed assessment of the influence of each type of thyroid dysfunction on biochemical parameters in different anovulatory conditions.

### 2.4 Statistical analysis

The analysis of quantitative variables was conducted by calculating descriptive statistics, which included the mean, standard deviation, median, quartiles, and minimum and maximum values. Qualitative variables were analyzed through the determination of absolute frequencies and percentages for all potential values. The assessment of normality was carried out using the Shapiro-Wilk test, with further verification through histograms and quantile-quantile plots. Intergroup comparisons of categorical variables employed Pearson’s chi-square test. For quantitative variables exhibiting a normal distribution, one-way analysis of variance (ANOVA) or t-tests were utilized, while the Kruskal-Wallis test or Wilcoxon test was applied to those not conforming to a normal distribution. Correlations between selected quantitative variables were evaluated using Pearson, Spearman, Kendall, and Point-Biserial correlation coefficients. Variables indicative of inflammatory status underwent logarithmic transformation, and those achieving normal distribution were incorporated into a logistic regression model. A significance level of p < 0.05 was established for all statistical analyses, which were performed using R software version 4.4.1 (27).

## 3 Results

The study involved 158 women with normogonadotropic anovulation, comprising 17 with HPOD and 141 with PCOS, who were further categorized into phenotypes A (n=82), B (n=23), and D (n=36). Detailed demographic, anthropometric, clinical, and biochemical data for all research groups were collected. The database was made publicly available through the Harvard Dataverse at https://doi.org/10.7910/DVN/CSU6WA. Significant intergroup differences were identified in several parameters that were characteristic of specific diagnoses, as detailed in **Table 1**. No intergroup differences in the prevalence of thyroid dysfunction were observed, as demonstrated in **Table 2**. In the next steps, the concentrations of selected inflammatory parameters were evaluated and compared among women with and without SCH and TAI across the entire cohort and within each study arm. A number of statistically significant differences in the values of the examined parameters were established, as detailed in **Table 3**.

**Table 1:** The comparative characteristics of the studied subpopulations of women with respect to selected demographic, anthropometric, clinical, and biochemical parameters.

Parameter		HPOD (N = 17)	PCOS A (N = 82)	PCOS B (N = 23)	PCOS D (N = 36)	Total (N = 158)	*p*
Age [years]	Mean (SD)	28.29 (6.89)	25.18 (5.28)	26.13 (5.32)	25.89 (4.47)	25.82 (5.34)	0.257
	Median (quartiles)	28 (22-35)	24 (21-28)	27 (22-29.5)	26 (22.75-28)	25 (22-29)	
	Range	19-42	18-44	18-39	18-36	18-44	
Weight [kg]	Mean (SD)	60.47 (6.1)	69.21 (20.33)	72.61 (15.97)	64.25 (17)	67.64 (18.17)	0.125
	Median (quartiles)	60 (57-65)	63 (55-81.5)	70 (58-89)	57 (53.75-70.75)	61.5 (55.25-74.5)	
	Range	50-69	46-130	53-95	46-115	46-130	
Height [cm]	Mean (SD)	164.53 (6.39)	165.12 (5.51)	164.04 (6.36)	165.79 (5.01)	165.05 (5.6)	0.678
	Median (quartiles)	164 (160-167)	164 (161.25-169)	164 (158-168.5)	165 (163-169)	164 (160.25-169)	
	Range	153-180	153-175	154-176	155-175	153-180	
BMI [kg/m^2^]	Mean (SD)	22.37 (2.3)	25.27 (6.82)	27.1 (6.32)	23.4 (6.17)	24.8 (6.37)	0.056
	Median (quartiles)	22.58 (20.68-24.38)	22.79 (20.23-29.75)	24.91 (21.63-32.49)	21.25 (19.36-24.6)	22.68 (19.86-28.26)	
	Range	17.93-26.04	17.5-42.45	18.72-37.32	17.31-41.24	17.31-42.45	
mFG score [n]	Mean (SD)	3.41 (2.03)	6.28 (5.83)	7.57 (5.27)	3.83 (2.06)	5.6 (4.98)	0.013*
	Median (quartiles)	3 (2-5)	4 (2.25-8)	8 (3-10.5)	4 (2-6)	4 (2-7)	
	Range	0-6	0-32	0-18	0-7	0-32	
Average menstrual cycle length [n]	Mean (SD)	73.53 (85.67)	74.27 (82.12)	53.3 (37.84)	82.36 (84.15)	72.98 (78)	0.425
	Median (quartiles)	37 (36-55)	45 (33-87)	40 (31-57)	43.75 (36.88-81.5)	45 (33.5-75.75)	
	Range	19.5-364	21-619	24.5-180	27-364	19.5-619	
Pregnancies [n]	Mean (SD)	0.29 (0.69)	0.06 (0.36)	0.26 (0.75)	0 (0)	0.1 (0.45)	0.022*
	Median (quartiles)	0 (0-0)	0 (0-0)	0 (0-0)	0 (0-0)	0 (0-0)	
	Range	0-2	0-3	0-3	0-0	0-3	
Deliveries [n]	Mean (SD)	0.29 (0.69)	0.05 (0.27)	0.22 (0.74)	0 (0)	0.09 (0.41)	0.037*
	Median (quartiles)	0 (0-0)	0 (0-0)	0 (0-0)	0 (0-0)	0 (0-0)	
	Range	0-2	0-2	0-3	0-0	0-3	
Miscarriages [n]	Mean (SD)	0 (0)	0.01 (0.11)	0.04 (0.21)	0 (0)	0.01 (0.11)	0.491
	Median (quartiles)	0 (0-0)	0 (0-0)	0 (0-0)	0 (0-0)	0 (0-0)	
	Range	0-0	0-1	0-1	0-0	0-1	
Right ovary volume [ml]	Mean (SD)	12.3 (7.81)	13.87 (8.97)	9.5 (5.74)	11.93 (3.65)	12.62 (7.6)	0.006*
	Median (quartiles)	9.72 (6.24-13.63)	12.63 (10.32-15.52)	8.51 (5.64-11.37)	11.41 (9.23-14.22)	11.32 (8.97-15.26)	
	Range	3.98-29.39	3.06-82.05	2.2-22.34	5.88-20.85	2.2-82.05	
Left ovary volume [ml]	Mean (SD)	6.3 (2.54)	14.6 (18.89)	8.06 (5.54)	10.47 (4.02)	11.81 (14.23)	<0.001*
	Median (quartiles)	5.59 (5-6.87)	11.42 (8.45-14.53)	6.7 (4.93-8.58)	10.12 (7.22-12.34)	10.12 (6.31-13.33)	
	Range	3.87-14.83	4.1-169.8	2.1-24.6	4.4-19.71	2.1-169.8	
AMH [pmol/l]	Mean (SD)	18.24 (7.39)	56.36 (32.56)	31.8 (11.35)	45.53 (26)	46.22 (29.84)	<0.001*
	Median (quartiles)	18.4 (11.9-22.6)	48.89 (31.13-66.85)	32.3 (24.35-36.25)	40.75 (27.4-51.33)	36.95 (27.5-54.83)	
	Range	6.71-34.5	12.9-164	11.3-65.2	15.3-119	6.71-164	
FSH [mIU/ml]	Mean (SD)	4.85 (1.64)	5.77 (1.8)	5.94 (1.71)	4.74 (1.86)	5.46 (1.83)	0.009*
	Median (quartiles)	4.94 (4.04-5.88)	5.86 (4.52-6.83)	5.8 (4.9-6.96)	4.62 (3.24-6.03)	5.58 (4.17-6.6)	
	Range	2.22-8.17	1.77-10.1	2.34-9.74	2.11-8.69	1.77-10.1	
LH [mIU/ml]	Mean (SD)	7.46 (6.36)	16.09 (10.98)	11.4 (6.08)	8.57 (5.77)	12.77 (9.59)	<0.001*
	Median (quartiles)	5.83 (3.8-8.77)	14.15 (8.2-20.93)	11 (6.96-15)	7 (4.28-10.83)	10.35 (6.67-16.6)	
	Range	1.73-29.6	2.86-70.2	3.44-30.8	1.82-23.6	1.73-70.2	
Estradiol [pmol/l]	Mean (SD)	429.48 (375.2)	369.35 (296.8)	389.83 (294.8)	296.55 (281.05)	362.21 (301.99)	0.275
	Median (quartiles)	448 (100-591)	250 (190.5-396.75)	297 (177.5-487.5)	229 (137-377.25)	251.5 (169-434.25)	
	Range	64.5-1443	78.9-1505	90-1306	51-1587	51-1587	
Prolactin [µIU/ml]	Mean (SD)	255.88 (121.78)	341.77 (109.12)	313.09 (109.14)	289.19 (116.27)	316.37 (115.07)	0.016*
	Median (quartiles)	251 (157-314)	353.5 (255.25-444.5)	316 (231-407.5)	265 (218.5-385.25)	316 (233.25-419.75)	
	Range	103-496	73-496	101-465	54-495	54-496	
Testosterone [nmol/l]	Mean (SD)	1.14 (0.34)	2.2 (0.63)	2.14 (0.74)	1.12 (0.26)	1.83 (0.75)	<0.001*
	Median (quartiles)	1.05 (1-1.39)	2.17 (1.8-2.53)	1.9 (1.7-2.3)	1.15 (1-1.23)	1.79 (1.22-2.33)	
	Range	0.36-1.67	0.52-4.1	1.22-4.07	0.47-1.61	0.36-4.1	
SHBG [nmol/l]	Mean (SD)	57.04 (23.45)	54.49 (33.3)	50.56 (27.03)	62.47 (28.09)	56.01 (30.36)	0.213
	Median (quartiles)	53.5 (44.5-63.1)	46.55 (28.6-69.8)	39.1 (32.95-70.5)	61.85 (40.08-86.23)	50.9 (33.4-72.5)	
	Range	27.6-124	10.2-196	12-116	17.7-128	10.2-196	
FAI [n]	Mean (SD)	2.16 (0.77)	5.58 (4.07)	5.41 (2.93)	2.14 (1.02)	4.41 (3.55)	<0.001*
	Median (quartiles)	2.01 (1.77-2.25)	4.51 (2.95-6.97)	4.54 (2.78-7.61)	1.92 (1.33-2.52)	3.24 (2.09-5.73)	
	Range	1.13-3.75	1.22-26.47	1.89-10.77	0.9-4.85	0.9-26.47	
DHEA-S [µmol/l]	Mean (SD)	7.04 (1.99)	9 (3.04)	9.11 (2.96)	6.56 (2.02)	8.25 (2.92)	<0.001*
	Median (quartiles)	6.22 (5.73-8.46)	8.64 (6.87-10.7)	8.75 (7.36-10.35)	dwq6.5 (5.09-8.5)	8.28 (6.08-9.9)	
	Range	4.38-11.3	2.82-16.6	2.01-15.2	2.82-10.4	2.01-16.6	
TSH [µIU/ml]	Mean (SD)	2.3 (1.72)	2.52 (1.21)	2.28 (1.22)	2.72 (1.38)	2.51 (1.31)	0.331
	Median (quartiles)	1.73 (1.2-2.49)	2.27 (1.59-3.47)	2.15 (1.34-3.13)	2.45 (1.85-3.59)	2.16 (1.58-3.47)	
	Range	0.65-6.57	0.85-6.02	0.24-4.98	0.76-6.5	0.24-6.57	
FT3 [pmol/l]	Mean (SD)	4.69 (0.97)	5.55 (0.72)	5.67 (0.58)	5.1 (0.91)	5.37 (0.83)	<0.001*
	Median (quartiles)	4.68 (3.79-5.38)	5.54 (5.1-6.1)	5.8 (5.44-5.95)	5.29 (4.43-5.62)	5.47 (4.84-5.98)	
	Range	3.24-6.09	3.14-6.77	4.09-6.8	3.28-6.76	3.14-6.8	
FT4 [pmol/l]	Mean (SD)	14.84 (2.56)	16.12 (2.07)	16.98 (2.26)	15.21 (2)	15.9 (2.22)	0.003*
	Median (quartiles)	14 (12.8-16.5)	16.2 (15-17.1)	16.7 (15.25-18.35)	14.8 (13.7-16.55)	15.9 (14.3-17.18)	
	Range	12.1-21.2	12.1-21.5	12.9-21.6	12.1-19.3	(12.1-21.6)	
TPOAb [IU/ml]	Mean (SD)	67.12 (109.96)	32.62 (80.9)	30.73 (39.38)	57.61 (138.73)	41.75 (96.47)	0.493
	Median (quartiles)	11.3 (8.99-85.1)	9.16 (8.99-16.7)	11.2 (8.99-26.25)	9.57 (8.99-19.78)	10.07 (8.99-19.45)	
	Range	8.99-437	8.99-600.1	8.99-150	8.99-600.1	(8.99-600.1)	
TGAb [IU/ml]	Mean (SD)	93.25 (112.88)	59.43 (80.79)	96.15 (143.89)	108.08 (153.24)	79.5 (115.16)	0.326
	Median (quartiles)	19.2 (15-150)	16.1 (14.73-51.88)	19.5 (15.1-120.55)	19.7 (15.15-132.25)	17.65 (14.8-114.5)	
	Range	13.3-323	11.5-297	13.8-581	12.7-771	(11.5-771)	
Fasting glucose [mmol/l]	Mean (SD)	4.78 (0.36)	4.83 (0.55)	4.82 (0.31)	4.68 (0.6)	4.79 (0.51)	0.325
	Median (quartiles)	4.68 (4.6-5.01)	4.79 (4.49-5.05)	4.8 (4.68-5.03)	4.65 (4.26-4.93)	4.75 (4.49-5.03)	
	Range	4.22-5.55	3.78-6.9	4.05-5.22	3.66-6.3	3.66-6.9	
120’ 75OGTT glucose [mmol/l]	Mean (SD)	5.85 (1.69)	5.4 (1.4)	5.85 (1.06)	5.16 (1.19)	5.46 (1.35)	0.123
	Median (quartiles)	5.86 (4.8-6.13)	5.2 (4.3-6.29)	5.77 (5.13-6.58)	5.15 (4.15-5.91)	5.34 (4.34-6.31)	
	Range	3.67-11.22	2.72-9.01	3.71-7.56	2.97-7.38	2.72-11.22	
Fasting insulin [uU/ml]	Mean (SD)	7.35 (3.6)	11.34 (8.24)	13.75 (9.33)	8.63 (6.02)	10.65 (7.77)	0.007*
	Median (quartiles)	6.28 (5.24-8.35)	8.1 (5.9-15.1)	10.4 (8.47-18.75)	6.34 (5.1-9.21)	7.99 (5.48-12.48)	
	Range	3.28-16	1.96-36.5	4.36-40.9	2.2-25.8	1.96-40.9	
120’ 75OGTT insulin [uU/ml]	Mean (SD)	58.75 (35.88)	66.9 (51.3)	69.48 (42.38)	52.21 (32.27)	63.05 (44.9)	0.511
	Median (quartiles)	44.8 (36.4-76.5)	47.75 (33.5-86)	59.9 (35.9-88.55)	43 (33.05-60.38)	47.3 (33.35-84.78)	
	Range	18.1-148	10.3-268	17.4-177	14.4-143	10.3-268	
HOMA-IR [n]	Mean (SD)	1.58 (0.86)	2.49 (1.92)	2.95 (1.98)	1.87 (1.49)	2.32 (1.79)	0.008*
	Median (quartiles)	1.37 (1.07-1.69)	1.74 (1.17-3.19)	2.1 (1.8-4.2)	1.28 (0.95-2)	1.65 (1.13-2.78)	
	Range	0.68-3.94	0.39-7.8	0.8-8.36	0.37-6.86	0.37-8.36	
Total cholesterol [mmol/l]	Mean (SD)	4.72 (0.64)	4.47 (0.74)	4.4 (0.72)	4.63 (0.73)	4.53 (0.73)	0.375
	Median (quartiles)	4.6 (4.4-5.3)	4.4 (4.1-4.9)	4.3 (3.95-4.75)	4.55 (4-5.13)	4.5 (4.1-5)	
	Range	3.4-6	2.4-6.6	3.2-6	3.6-6.1	2.4-6.6	
HDL-cholesterol [mmol/l]	Mean (SD)	1.91 (0.55)	1.68 (0.38)	1.61 (0.35)	1.84 (0.43)	1.73 (0.42)	0.034*
	Median (quartiles)	1.88 (1.5-2.38)	1.6 (1.41-2.02)	1.62 (1.39-1.82)	1.79 (1.56-2.08)	1.69 (1.45-2.03)	
	Range	0.99-3	0.91-2.57	0.96-2.31	1.05-2.83	0.91-3	
LDL- cholesterol [mmol/l]	Mean (SD)	2.47 (0.63)	2.39 (0.66)	2.39 (0.54)	2.41 (0.59)	2.41 (0.62)	0.978
	Median (quartiles)	2.6 (1.9-2.8)	2.4 (2-2.78)	2.4 (1.9-2.7)	2.4 (2.08-2.73)	2.4 (2-2.8)	
	Range	1.2-3.3	0.7-4	1.6-3.4	1.3-4	0.7-4	
Triglycerides [mmol/l]	Mean (SD)	0.73 (0.38)	0.84 (0.42)	0.86 (0.32)	0.81 (0.4)	0.83 (0.4)	0.376
	Median (quartiles)	0.63 (0.56-0.72)	0.73 (0.56-1.01)	0.81 (0.62-0.93)	0.7 (0.52-0.92)	0.72 (0.56-0.96)	
	Range	0.4-2.04	0.33-2.29	0.41-1.88	0.38-2.29	0.33-2.29	
AST [U/l]	Mean (SD)	20.65 (5.91)	23.42 (8.13)	22.48 (5.3)	22.86 (7.01)	22.85 (7.3)	0.394
	Median (quartiles)	20 (17-22)	22 (18-26.75)	21 (19.5-24)	21.5 (18.75-25.25)	21 (18.25-25)	
	Range	14-40	14-63	16-37	14-55	14-63	
ALT [U/l]	Mean (SD)	18.65 (14.2)	22.05 (14.39)	23.7 (13.6)	19.72 (12.07)	21.39 (13.72)	0.181
	Median (quartiles)	14 (13-18)	16.5 (14-25)	19 (16-27)	17 (14-20.5)	17 (14-24)	
	Range	9-71	4.99-85	10-63	6-77	4.99-85	
Vitamin D [ng/ml]	Mean (SD)	31.34 (14.25)	27.11 (10.62)	30.54 (12.66)	27.51 (9.99)	28.16 (11.23)	0.399
	Median (quartiles)	28 (22.5-34.3)	24.15 (19.4-31.95)	30.1 (21.35-35.4)	27.2 (19.78-33.55)	26.05 (20.4-33.75)	
	Range	17.7-76.4	9.18-64.6	10.1-61.3	10.2-46.6	9.18-76.4	
CRP [mg/l]	Mean (SD)	1.24 (1.11)	2.39 (3.83)	2.99 (3.41)	1.57 (2.39)	2.17 (3.3)	0.067
	Median (quartiles)	0.61 (0.59-1.26)	0.99 (0.59-2.86)	1.65 (0.59-3.94)	0.59 (0.59-1.25)	0.76 (0.59-2.3)	
	Range	0.59-4.31	0.59-25.7	0.59-14	0.59-13.5	0.59-25.7	
IL-6 [pg/ml]	Mean (SD)	2.06 (1.12)	2.77 (4.13)	2.07 (0.83)	2.3 (2.48)	2.48 (3.24)	0.144
	Median (quartiles)	1.49 (1.49-1.75)	1.82 (1.49-2.67)	1.67 (1.49-2.21)	1.49 (1.49-2.03)	1.68 (1.49-2.44)	
	Range	1.49-5.06	1.49-38.1	1.49-4.31	1.49-16.2	1.49-38.1	
IL-10 [pg/ml]	Mean (SD)	18.48 (44.64)	2.38 (3.66)	4.85 (8.19)	6.7 (25.05)	5.46 (19.57)	0.174
	Median (quartiles)	2.07 (0.7-7.39)	1.09 (0.51-2.8)	0.85 (0.56-5.46)	1.7 (0.69-3.9)	1.14 (0.58-3.26)	
	Range	0.3-174.58	0-18.91	0.29-36.76	0.27-152.13	0-174.58	
TNF-α [pg/ml]	Mean (SD)	4.67 (2.04)	6.03 (3.55)	5.43 (1.68)	5.85 (2.93)	5.76 (3.07)	0.532
	Median (quartiles)	4.79 (3.34-5.84)	5.11 (3.95-7)	5.11 (4.22-6.26)	5.4 (3.8-6.85)	5.18 (3.86-6.82)	
	Range	0-8.39	0-25.8	3.31-9.38	1.29-14.29	0-25.8	
IL-1β [pg/ml]	Mean (SD)	0.27 (0.19)	0.29 (0.31)	0.38 (0.46)	0.33 (0.33)	0.31 (0.33)	0.748
	Median (quartiles)	0.19 (0.12-0.36)	0.15 (0.07-0.45)	0.18 (0.1-0.47)	0.23 (0.08-0.49)	0.18 (0.08-0.45)	
	Range	0.06-0.67	0.02-1.71	0.06-1.8	0.02-1.35	0.02-1.8	
IL-6/IL-10 [n]	Mean (SD)	1.64 (2.02)	6.09 (30.22)	2.5 (2.58)	1.98 (1.92)	4.13 (21.72)	0.097
	Median (quartiles)	0.72 (0.22-2.48)	2.09 (0.63-4.04)	2.51 (0.3-3.43)	1.48 (0.45-3.11)	1.76 (0.5-3.52)	
	Range	0.01-7.93	0.09-272.14	0.04-8.29	0.01-7.3	0.01-272.14	
TNF-α/IL-10 [n]	Mean (SD)	4.09 (4.22)	6.29 (5.69)	5.55 (4.84)	4.97 (4.77)	5.64 (5.23)	0.215
	Median (quartiles)	2.39 (0.59-5.02)	5.06 (2.55-8.84)	3.89 (0.92-9.91)	3.46 (1.47-6.74)	4.27 (1.66-8.62)	
	Range	0-12.5	0-32.86	0.24-14.43	0.02-18.43	0-32.86	
IL-1β/IL-10 [n]	Mean (SD)	0.15 (0.15)	0.2 (0.25)	0.31 (0.67)	0.17 (0.16)	0.2 (0.32)	0.769
	Median (quartiles)	0.1 (0.07-0.18)	0.13 (0.07-0.24)	0.14 (0.08-0.22)	0.13 (0.06-0.21)	0.13 (0.07-0.23)	
	Range	0-0.54	0.01-1.86	0.01-3.27	0-0.79	0-3.27	
CRP/SHBG [n]	Mean (SD)	0.03 (0.03)	0.08 (0.18)	0.09 (0.12)	0.04 (0.06)	0.07 (0.14)	0.073
	Median (quartiles)	0.02 (0.01-0.02)	0.02 (0.01-0.09)	0.04 (0.01-0.12)	0.01 (0.01-0.03)	0.02 (0.01-0.08)	
	Range	0.01-0.1	0-1.29	0.01-0.41	0.01-0.31	0-1.29	

p, parametric one-way analysis of variance (ANOVA) or nonparametric Kruskal-Wallis test; * statistically significant (p < 0.05); PCOS, polycystic ovary syndrome; HPOD, hypothalamic-pituitary-ovarian dysfunction; SD, standard deviation; BMI, body mass index; PCOM, polycystic ovarian morphology; AMH, anti-Müllerian hormone; FSH, follicle-stimulating hormone; LH, luteinizing hormone; SHBG, sex hormone binding globulin; FAI, free androgen index; DHEA-S, dehydroepiandrosterone sulfate; TSH, thyroid-stimulating hormone; fT3, free triiodothyronine; fT4, free thyroxine; TPOAb, thyroid peroxidase antibodies; TGAb, thyroglobulin antibodies; OGTT, 75-g oral glucose tolerance test; HOMA-IR, homeostasis model assessment for insulin resistance; AST, aspartate transaminase; ALT, alanine transaminase; CRP, C-reactive protein; TNF-α, tumor necrosis factor-α.

**Table 2:** The comparison of the frequency of subclinical hypothyroidism (SCH) and thyroid autoimmunity (TAI) among women with hypothalamic-pituitary-ovarian dysfunction (HPOD) and specific phenotypes of polycystic ovary syndrome (PCOS).

Parameter		HPOD (N = 17)	PCOS A (N = 82)	PCOS B (N = 23)	PCOS D (N = 36)	Total (N = 158)	*p*
SCH	No SCH	13 (76.5%)	44 (53.7%)	13 (56.5%)	19 (52.8%)	89 (56.3%)	0.358
	SCH	4 (23.5%)	38 (46.3%)	10 (43.5%)	17 (47.2%)	69 (43.7)	
TPOAb	Negative	10 (58.8%)	69 (84.1%)	17 (73.9%)	28 (77.8%)	124 (78.5%)	0.125
	Positive	7 (41.2%)	13 (15.9%)	6 (26.1%)	8 (22.2%)	34 (21.5%)	
TGAb	Negative	11 (64.7%)	67 (81.7%)	17 (73.9%)	24 (66.7%)	119 (75.3%)	0.230
	Positive	6 (35.3%)	15 (18.3%)	6 (26.1%)	12 (33.3%)	39 (24.7%)	
ATA (TPOAb, TGAb)	Both negative	8 (47.1%)	61 (74.4%)	13 (56.5%)	21 (58.3%)	103 (65.2%)	0.071
	At least one positive	9 (52.9%)	21 (25.6%)	10 (43.5%)	15 (41.7%)	55 (34.8%)	

p, Pearson’s chi-squared test; * statistically significant (p < 0.05); PCOS, polycystic ovary syndrome; HPOD, hypothalamic-pituitary-ovarian dysfunction; SCH, subclinical hypothyroidism; ATA, anti-thyroid antibodies; TPOAb, thyroid peroxidase antibodies; TGAb, thyroglobulin antibodies.

**Table 3:** The comparative analysis of the concentrations of inflammatory parameters in women across the entire cohort and within individual study arms, with respect to the presence or absence of subclinical hypothyroidism (SCH), as well as regarding thyroid autoimmunity (TAI).

		Entire cohort						PCOS						HPOD					
Parameter		SCH			TAI			SCH			TAI			SCH			TAI		
		No	Yes	*p*	No	Yes	*p*	No	*Yes*	*p*	*No*	*Yes*	*p*	*No*	*Yes*	*p*	*No*	*Yes*	*p*
CRP [mg/l]	n	89	69	0.017*	103	55	0.458	78	63	0.056	94	47	0.284	13	4	0.189	8	9	0.104
	Mean (SD)	1.74 (2.86)	2.71 (3.74)		2.31 (3.69)	1.9 (2.4)		1.91 (3.06)	2.74 (3.87)		2.45 (3.84)	1.93 (2.54)		0.95 (0.71)	2.18 (1.73)		0.71 (0.18)	1.71 (1.38)	
	Median (quartiles)	0.61 (0.59-1.54)	1.27 (0.59-3.69)		0.96 (0.59-2.22)	0.59 (0.59-2.52)		0.61 (0.59-1.67)	1.27 (0.59-3.54)		0.99 (0.59-2.97)	0.59 (0.59-2.17)		0.59 (0.59-0.99)	1.9 (0.87-3.21)		0.59 (0.59-0.82)	1.26 (0.59-2.84)	
	Range	(0.59-20)	(0.59-25.7)		(0.59-25.7)	(0.59-14)		(0.59-20)	(0.59-25.7)		(0.59-25.7)	(0.59-14)		(0.59-3.08)	(0.59-4.31)		(0.59-0.99)	(0.59-4.31)	
IL-6 [pg/ml]	n	89	69	0.654	103	55	0.002*	78	63	0.966	94	47	<0.001*	13	4	0.947	8	9	1.000
	Mean (SD)	2.55 (3.95)	2.39 (2.01)		2.42 (1.73)	2.6 (4.98)		2.65 (4.20)	2.38 (2.06)		2.46 (1.77)	2.66 (5.37)		1.96 (0.91)	2.38 (1.79)		1.99 (1.24)	2.12 (1.07)	
	Median (quartiles)	1.63 (1.49-2.45)	1.69 (1.49-2.39)		1.83 (1.49-2.67)	1.49 (1.49-1.92)		1.68 (1.49-2.67)	1.70 (1.49-2.37)		2.00 (1.49-2.68)	1.49 (1.49-1.86)		1.49 (1.49-1.75)	1.49 (1.49-2.38)		1.49 (1.49-1.71)	1.49 (1.49-2.38)	
	Range	(1.49-38.1)	(1.49-16.2)		(1.49-16.2)	(1.49-38.1)		(1.49-38.1)	(1.49-16.2)		(1.49-16.2)	(1.49-38.1)		(1.49-4.42)	(1.49-5.06)		(1.49-5.06)	(1.49-4.42)	
IL-10 [pg/ml]	n	89	69	0.023*	103	55	0.014*	78	63	0.009*	94	47	0.005*	13	4	0.871	8	9	0.673
	Mean (SD)	5.46 (20.36)	5.46 (18.65)		6.55 (24.02)	3.41 (4.1)		2.34 (3.68)	5.81 (19.49)		4.24 (16.20)	3.19 (3.50)		23.61 (50.36)	1.81 (0.53)		33.99 (63.15)	4.7 (6.42)	
	Median (quartiles)	0.89 (0.51-3.16)	1.91 (0.77-3.4)		0.89 (0.52-2.87)	2.03 (0.86-3.73)		0.87 (0.5-2.19)	1.91 (0.7-4.08)		0.87 (0.50-2.60)	1.96 (1.09-3.73)		2.9 (0.67-11.34)	1.775 (1.43-2.16)		1.375 (0.91-28.74)	2.42 (0.6-5.5)	
	Range	(0-174.58)	(0.3-152.13)		(0.18-174.58)	(0-20.58)		(0-18.91)	(0.3-152.13)		(0.18-152.13)	(0-18.91)		(0.3-174.58)	(1.27-2.42)		(0.67-174.58)	(0.3-20.58)	
TNF-α [pg/ml]	n	89	69	0.013*	103	55	0.036*	78	63	0.045*	94	47	0.028*	13	4	0.469	8	9	0.229
	Mean (SD)	5.18 (2.28)	6.5 (3.75)		5.37 (2.63)	6.48 (3.68)		5.33 (2.27)	6.57 (3.90)		5.48 (2.65)	6.71 (3.87)		4.49 (2.19)	5.25 (1.57)		4.01 (2.26)	5.26 (1.73)	
	Median (quartiles)	4.79 (3.71-6.06)	5.45 (4.54-7.61)		4.97 (3.73-6.32)	5.56 (4.48-7.63)		4.99 (3.86-6.22)	5.45 (4.44-7.93)		5.07 (3.83-6.42)	5.59 (4.52-8.26)		4.47 (3.26-5.84)	4.94 (4.61-5.59)		4.065 (2.98-5.19)	4.91 (3.69-6.25)	
	Range	(0-13.15)	(0-25.8)		(0-16.41)	(1.68-25.8)		(0-13.15)	(0-25.8)		(0-16.41)	(1.68-25.8)		(0-8.39)	(3.69-7.43)		(0-7.43)	(3.26-8.39)	
IL-1β [pg/ml]	n	89	69	0.042*	103	55	<0.001*	78	63	0.017*	94	47	<0.001*	13	4	0.996	8	9	0.080
	Mean (SD)	0.28 (0.33)	0.35 (0.33)		0.25 (0.3)	0.42 (0.36)		0.28 (0.34)	0.37 (0.34)		0.26 (0.31)	0.44 (0.38)		0.27 (0.19)	0.27 (0.21)		0.18 (0.1)	0.34 (0.23)	
	Median (quartiles)	0.14 (0.07-0.38)	0.24 (0.1-0.5)		0.12 (0.06-0.38)	0.35 (0.17-0.57)		0.12 (0.07-0.38)	0.28 (0.11-0.51)		0.12 (0.06-0.38)	0.35 (0.17-0.57)		0.19 (0.12-0.36)	0.2 (0.15-0.32)		0.16 (0.12-0.24)	0.32 (0.17-0.56)	
	Range	(0.02-1.8)	(0.02-1.71)		(0.02-1.71)	(0.06-1.8)		(0.02-1.8)	(0.02-1.71)		(0.02-1.71)	(0.06-1.8)		(0.06-0.67)	(0.1-0.57)		(0.06-0.36)	(0.06-0.67)	
IL-6/IL-10	n	87	69	0.205	103	53	<0.001*	76	63	0.064	94	45	<0.001*	13	4	0.477	8	9	0.815
	Mean (SD)	5.69 (28.99)	2.16 (2.32)		2.93 (2.5)	6.45 (37.23)		6.29 (30.99)	2.19 (2.38)		3.08 (2.53)	7.24 (40.40)		1.66 (2.18)	1.58 (1.61)		1.45 (1.43)	1.81 (2.51)	
	Median (quartiles)	2.427 (0.58-3.43)	0.837 (0.46-3.7)		2.636 (0.62-4.04)	0.72 (0.44-2.29)		2.56 (0.72-3.91)	0.84 (0.45-3.64)		2.95 (0.67-4.14)	0.73 (0.42-2.09)		0.6 (0.2-2.48)	0.863 (0.69-1.75)		1.366 (0.1-2.29)	0.616 (0.51-2.48)	
	Range	(0.01-272.14)	(0.01-11.52)		(0.01-11.52)	(0.14-272.14)		(0.09-272.14)	(0.01-11.52)		(0.01-11.52)	(0.14-272.14)		(0.01-7.93)	(0.62-3.98)		(0.01-3.98)	(0.2-7.93)	
TNF-α/IL-10	n	87	69	0.250	103	53	0.025*	76	63	0.139	94	45	0.011*	13	4	0.871	8	9	0.606
	Mean (SD)	6.25 (5.96)	4.87 (4.05)		6.14 (5.04)	4.67 (5.52)		6.53 (6.05)	4.98 (4.19)		6.42 (5.11)	4.59 (5.61)		4.37 (4.78)	3.19 (1.55)		3.45 (3.12)	4.66 (5.13)	
	Median (quartiles)	4.888 (1.73-9.38)	3.464 (1.59-7.57)		5.034 (2.18-9.38)	2.864 (1.31-6.06)		5.17 (2.59-9.38)	3.46 (1.51-8.06)		5.61 (2.63-9.81)	3.02 (1.31-5.24)		2.393 (0.41-8.72)	2.971 (1.97-4.19)		4.342 (0.21-4.92)	2.029 (0.61-9.72)	
	Range	(0-32.86)	(0-14.43)		(0-30.22)	(0.4-32.86)		(0-32.86)	(0-14.43)		(0-30.22)	(0.40-32.86)		(0-12.5)	(1.78-5.02)		(0-8.72)	(0.41-12.5)	
IL-1 β/IL-10	n	87	69	0.899	103	53	0.332	76	63	0.929	94	45	0.514	13	4	0.871	8	9	0.277
	Mean (SD)	0.22 (0.4)	0.18 (0.18)		0.17 (0.17)	0.26 (0.5)		0.23 (0.43)	0.19 (0.19)		0.18 (0.17)	0.28 (0.54)		0.15 (0.16)	0.15 (0.1)		0.13 (0.18)	0.17 (0.12)	
	Median (quartiles)	0.125 (0.06-0.23)	0.124 (0.08-0.21)		0.123 (0.07-0.2)	0.133 (0.08-0.23)		0.13 (0.06-0.23)	0.12 (0.08-0.21)		0.13 (0.07-0.21)	0.13 (0.08-0.23)		0.102 (0.02-0.15)	0.126 (0.07-0.21)		0.077 (0.02-0.14)	0.12 (0.09-0.28)	
	Range	(0-3.27)	(0-0.91)		(0-0.91)	(0.02-3.27)		(0.01-3.27)	(0-0.91)		(0.0007-0.91)	(0.02-3.27)		(0-0.54)	(0.07-0.28)		(0-0.54)	(0.02-0.37)	
CRP/SHBG	n	89	69	0.016*	103	55	0.168	78	63	0.013*	94	47	0.216	13	4	0.350	8	9	0.114
	Mean (SD)	0.05 (0.11)	0.09 (0.17)		0.08 (0.17)	0.05 (0.08)		0.06 (0.12)	0.09 (0.18)		0.09 (0.17)	0.05 (0.08)		0.02 (0.01)	0.05 (0.05)		0.01 (0)	0.04 (0.03)	
	Median (quartiles)	0.014 (0.01-0.04)	0.025 (0.01-0.11)		0.017 (0.01-0.09)	0.013 (0.01-0.06)		0.01 (0.01-0.04)	0.03 (0.01-0.11)		0.02 (0.01-0.10)	0.01 (0.01-0.06)		0.012 (0.01-0.02)	0.052 (0.02-0.09)		0.012 (0.01-0.02)	0.022 (0.01-0.04)	
	Range	(0-0.7)	(0-1.29)		(0-1.29)	(0.01-0.41)		(0-0.7)	(0-1.29)		(0.003-1.29)	(0.005-0.41)		(0.01-0.04)	(0.01-0.1)		(0.01-0.02)	(0.01-0.1)	

p, parametric Student’s T test or nonparametric Mann-Whitney U test; * statistically significant (p < 0.05); SD, standard deviation; PCOS, polycystic ovary syndrome; HPOD, hypothalamic-pituitary-ovarian dysfunction; SHBG, sex hormone binding globulin; CRP, C-reactive protein; TNF-α, tumor necrosis factor-α; ATA, anti-thyroid antibodies; TPOAb, thyroid peroxidase antibodies; TGAb, thyroglobulin antibodies.

The multiple regression analysis conducted to assess the impact of SCH and TAI on the concentrations of the examined inflammatory parameters, both in the entire cohort and in the individual study arms, yielded several significant findings, as evidenced in **Table 4**.

**Table 4:** Results of the logistic regression for analyzed inflammatory markers in relation to subclinical hypothyroidism (SCH) and thyroid autoimmunity (TAI).

Inflammatory parameter	Subpopulation	Entire cohort		PCOS		HPOD	
		β	*p*	β	*p*	β	*p*
IL-10 [pg/ml]	Subclinical hypothyroidism	0,622	0,903	NaN	NaN	<0,001	0,250
	Thyroid autoimmunity	0,038	0,442	3,095	0,721	<0,001	0,132
	Interaction	1,198	0,979	0,013	0,382	NaN	NaN
TNF-α [pg/ml]	Subclinical hypothyroidism	5,228	0,005*	4,441	0,018*	NaN	NaN
	Thyroid autoimmunity	4,596	0,018*	4,465	0,039*	NaN	NaN
	Interaction	0,597	0,615	0,867	0,899	0,016	0,072
IL-6/IL-10	Subclinical hypothyroidism	0,584	0,900	0,450	0,866	4,032	0,434
	Thyroid autoimmunity	NaN	NaN	NaN	NaN	2,805	0,397
	Interaction	0,000	0,281	0,000	0,251	0,057	0,259
TNF-α/IL-10	Subclinical hypothyroidism	0,298	0,237	0,196	0,132	3,881	0,715
	Thyroid autoimmunity	0,297	0,287	0,190	0,192	NaN	NaN
	Interaction	0,320	0,527	0,553	0,762	0,007	0,353
IL-1β/IL-10	Subclinical hypothyroidism	1,051	0,428	1,052	0,459	0,996	0,974
	Thyroid autoimmunity	1,205	0,009*	1,256	0,005*	1,036	0,700
	Interaction	0,780	0,026*	0,743	0,017*	1,013	0,943

p, general linear model; β, regression coefficient; * statistically significant (p < 0.05); PCOS, polycystic ovary syndrome; HPOD, hypothalamic-pituitary-ovarian dysfunction; TNF-α, tumor necrosis factor-α; NaN, not a number.

In the entire cohort, both SCH and TAI independently and significantly increased TNF-α concentrations (p=0.005, p=0.018, respectively). Additionally, TAI was associated with a significant increase in the IL-1β/IL-10 ratio (p=0.009), with a significant interaction effect observed between SCH and TAI for this parameter (p=0.026). In the PCOS arm, analogous associations were identified, with SCH and TAI exhibiting significant independent effects on TNF-α concentrations (p=0.018 and p=0.039, respectively), and TAI being linked to a significant increase in the IL-1β/IL-10 ratio (p=0.005), along with a significant interaction effect between SCH and TAI (p=0.017). Conversely, the HPOD arm showed no significant associations between SCH or TAI and the examined inflammatory markers (all p-values> 0.05).

The correlations between the concentrations of inflammatory parameters and the values of metabolic and ovarian indicators were subsequently examined in women with SCH and TAI within the entire cohort and within each study arm. Multiple significant correlations among the examined variables were established, as outlined in **Table 5**.

**Table 5:** Significant correlations between the concentrations of inflammatory parameters and the values of metabolic and ovarian indicators within the subpopulation with subclinical hypothyroidism (SCH) and thyroid autoimmunity (TAI) of the entire cohort and both study arms.

Inflammatory marker	Variable	Entire cohort						PCOS						HPOD					
		Total		SCH		TAI		Total		SCH		TAI		Total		SCH		TAI	
		r	*p*	r	*p*	r	*p*	r	*p*	r	*p*	r	*p*	r	*p*	r	*p*	r	*p*
CRP [mg/l]	Age [years]													0.375	0.049*				
	Weight [kg]	0.634	<0.001*	0.649	<0.001*	0.634	<0.001*	0.670	<0.001*	0.666	<0.001*	0.539	<0.001*						
	BMI [kg/m2]	0.711	<0.001*	0.731	<0.001*	0.711	<0.001*	0.740	<0.001*	0.740	<0.001*	0.699	<0.001*						
	Average menstrual cycle length [n]																	0.567	0.041*
	FSH [uIU/ml]					0.239	0.015*												
	Estradiol [pmol/l]			-0.283	0.018*					-0.284	0.022*					-0.283	0.018*		
	SHBG [nmol/l]	-0.510	<0.001*	-0.554	<0.001*	-0.482	<0.001*	-0.539	<0.001*	-0.545	<0.001*	-0.637	<0.001*			-0.554	<0.001*	-0.565	<0.001*
	FAI [n]	0.442	<0.001*	0.436	<0.001*	0.480	<0.001*	0.461	<0.001*	0.441	<0.001*	0.438	0.002*			0.436	<0.001*	0.372	0.005*
	TSH [uIU/ml]	0.207	0.009*			0.206	0.037*	0.181	0.032*										
	FT3 [pmol/l]	0.269	0.001*			0.273	0.005*	0.244	0.003*					0.479	0.011*				
	Fasting glucose [mmol/l]							0.168	0.046*										
	TPOAb [IU/ml]													0.426	0.033*				
	120’ 75OGTT glucose [mmol/l]	0.245	0.002*	0.277	0.021*			0.261	0.002*	0.305	0.014*	0.424	0.003*			0.277	0.021*	0.389	0.003*
	Fasting insulin [uU/ml]	0.588	<0.001*	0.586	<0.001*	0.638	<0.001*	0.626	<0.001*	0.618	<0.001*	0.595	<0.001*			0.586	<0.001*	0.492	<0.001*
	120’ 75OGTT insulin [uU/ml]	0.394	<0.001*	0.549	<0.001*	0.386	<0.001*	0.408	<0.001*	0.567	<0.001*	0.435	0.003*			0.549	<0.001*	0.422	0.001*
	HOMA-IR [n]	0.572	<0.001*	0.579	<0.001*	0.621	<0.001*	0.613	<0.001*	0.618	<0.001*	0.587	<0.001*			0.579	<0.001*	0.472	<0.001*
	HDL-cholesterol [mmol/l]	-0.523	<0.001*	-0.690	<0.001*	-0.586	<0.001*	-0.554	<0.001*	-0.673	<0.001*	-0.497	<0.001*			-0.690	<0.001*	-0.413	0.002*
	LDL-cholesterol [mmol/l]	0.237	0.003*	0.360	0.002*	0.259	0.008*	0.254	0.002*	0.323	0.009*					0.360	0.002*		
	Triglycerides [mmol/l]	0.486	<0.001*	0.560	<0.001*	0.546	<0.001*	0.507	<0.001*	0.590	<0.001*	0.438	0.002*			0.560	<0.001*	0.371	0.005*
	ALT [U/l]	0.338	<0.001*	0.378	0.001*	0.469	<0.001*	0.399	<0.001*	0.377	0.002*					0.378	0.001*		
	AST [U/l]					0.243	0.013*							-0.383	0.048*				
	Vitamin D [ng/ml]									-0.248	0.047*								
IL-6 [pg/ml]	Weight [kg]	0.345	<0.001*	0.302	0.012*	0.345	<0.001*	0.357	<0.001*	0.317	0.010*								
	BMI [kg/m2]	0.365	<0.001*	0.358	0.002*	0.365	<0.001*	0.390	<0.001*	0.371	0.002*								
	Average menstrual cycle length [n]																		
	Pregnancies [n]																		
	Deliveries [n]																		
	Testosterone [nmol/l]	0.164	0.040*																
	AMH [pmol/l]							-0.185	<0.001*			-0.367	0.012*						
	SHBG [nmol/l]	-0.183	0.022*			-0.206	0.037*	-0.205	<0.001*										
	FAI	0.247	0.002*			0.285	0.004*	0.265	<0.001*										
	TPOAb [IU/ml]							-0.169	0.045*										
	TGAb [IU/ml]	-0.214	0.007*					-0.234	0.005*										
	FT3 [pmol/l]	0.238	0.003*					0.207	<0.001*										
	120’ 75OGTT glucose [mmol/l]	0.169	0.034*			0.217	0.028*	0.169	0.045*										
	Fasting insulin [uU/ml]	0.371	<0.001*	0.406	0.001*	0.421	<0.001*	0.385	<0.001*	0.433	<0.001*					0.406	<0.001*		
	120’ 75OGTT insulin [uU/ml]	0.308	<0.001*	0.433	<0.001*			0.326	<0.001*	0.455	<0.001*					0.433	<0.001*		
	HOMA-IR [n]	0.368	<0.001*	0.410	<0.001*	0.417	<0.001*	0.378	<0.001*	0.430	<0.001*					0.410	<0.001*		
	Triglycerides [mmol/l]	0.237	0.003*	0.458	<0.001*	0.360	<0.001*	0.228	<0.001*	0.461	<0.001*					0.458	<0.001*		
	HDL-cholesterol [mmol/l]	-0.243	0.002*			-0.307	0.002*	-0.293	<0.001*	-0.321	0.009*								
	ALT [U/l]					0.204	0.039*	0.167	<0.001*										
	Vitamin D [ng/ml]	-0.175	0.028*	-0.245	0.042*	-0.262	0.007*							-0.391	0.047*	-0.245	0.042*		
IL-10 [pg/ml]	Testosterone [nmol/l]			-0.290	0.016*					-0.300	0.015*					-0.290	0.016*		
	TSH [uIU/ml]	0.185	0.020*	0.270	0.025*	0.219	0.026*	0.273	0.001*	0.266	0.032*					0.270	0.025*		
	120’ 75OGTT insulin [uU/ml]											0.355	0.016*						
	Triglycerides [mmol/l]							0.181	0.032*										
	ALT [U/l]													0.406	0.025*				
	Vitamin D [ng/ml]													0.549	0.024*				
TNF-α [pg/ml]	Weight [kg]			0.287	0.017*	0.209	0.008*	0.213	0.011*	0.284	0.022*								
	BMI [kg/m2]	0.246	0.002*	0.324	0.007*	0.246	0.002*	0.245	0.003*										
	AMH [pmol/l]	0.208	0.009*									0.496	<0.001*	0.549	0.022*			0.515	<0.001*
	SHBG [nmol/l]	-0.193	0.015*			-0.254	0.010*	-0.207	0.014*										
	FAI					0.207	0.036*												
	TSH [uIU/ml]	0.182	0.022*			0.350	<0.001*	0.191	0.023*	0.262	0.035*								
	TPOAb [IU/ml]	0.192	0.016*					0.223	0.008*			0.346	0.019*						
	FT3 [pmol/l]					0.282	0.004*							0.572	0.016*				
	120’ 75OGTT glucose [mmol/l]	0.223	0.005*	0.319	0.007*	0.213	0.031*	0.251	0.003*	0.340	0.006*					0.319	0.007*		
	Fasting insulin [uU/ml]					0.217	0.028*												
	120’ 75OGTT insulin [uU/ml]	0.222	0.005*	0.246	0.041*	0.246	0.012*	0.190	0.024*	0.252	0.043*			0.532	0.030*	0.246	0.041*		
	HOMA-IR	0.169	0.033*			0.221	0.025*												
	Total cholesterol [mmol/l]	0.172	0.031*					0.204	0.015*									0.300	0.026*
	HDL-cholesterol [mmol/l]	-0.208	0.009*	-0.275	0.022*	-0.254	0.010*	-0.196	0.020*	-0.303	0.014*					-0.275	0.022*		
	LDL-cholesterol [mmol/l]	0.225	0.004*					0.249	0.003*			0.333	0.024*					0.336	0.012*
	Triglycerides [mmol/l]	0.279	<0.001*	0.263	0.029*	0.236	0.016*	0.246	0.003*	0.270	0.030*					0.263	0.029*	0.292	0.030*
	DHEA-S [umol/l]													0.516	0.034*				
	ALT [U/l]	0.173	0.030*					0.182	0.031*			0.328	0.026*					0.334	0.013*
IL-1β [pg/ml]	Pregnancies [n]															0.967	0.033*		
	Deliveries [n]															0.967	0.033*		
	Estradiol [pmol/l]									0.270	0.030*								
	DHEA-S [umol/l]			-0.288	0.016*					-0.282	0.023*					-0.288	0.016*		
	TSH [uIU/ml]	0.195	0.014*	0.261	0.031*	0.331	0.001*	0.222	0.008*	0.276	0.026*					0.261	0.031*		
	TPOAb [IU/ml]	0.203	0.011*					0.208	0.013*									-0.281	0.037*
	TGAb [IU/ml]	0.340	<0.001*	0.263	0.029*			0.329	<0.001*	0.250	0.045*					0.263	0.029*		
	Fasting glucose [mmol/l]					0.225	0.022*												
	120’ 75OGTT glucose [mmol/l]	0.191	0.016*			0.194	0.049*	0.222	0.008*			0.323	0.029*						
	Fasting insulin [uU/ml]					0.262	0.007*												
	HOMA-IR					0.282	0.004*												
	120’ 75OGTT insulin [uU/ml]											0.325	0.028*						

r Spearman’s or Pearson’s correlation coefficient; * statistically significant (p < 0.05); PCOS, polycystic ovary syndrome; HPOD, hypothalamic-pituitary-ovarian dysfunction; BMI, body mass index; AMH, anti-Müllerian hormone; FSH, follicle-stimulating hormone; SHBG, sex hormone binding globulin; FAI, free androgen index; DHEA-S, dehydroepiandrosterone sulfate; TSH, thyroid-stimulating hormone; fT3, free triiodothyronine; TPOAb, thyroid peroxidase antibodies; TGAb, thyroglobulin antibodies; OGTT, 75-g oral glucose tolerance test; HOMA-IR, homeostasis model assessment for insulin resistance; AST, aspartate transaminase; ALT, alanine transaminase; CRP, C-reactive protein; TNF-α=tumor necrosis factor-α.

The analysis of selected metabolic and ovarian parameters in relation to inflammatory markers, SCH, and TAI across the entire study cohort and both study arms revealed significant associations, as detailed in **Table 6**. In the entire cohort, AMH concentrations demonstrated a significant inverse relationship with IL-10 in both univariate and multivariate models (p=0.011 and p=0.014, respectively), as well as in the multivariate model within the PCOS (p=0.014) and HPOD (p=0.014) arms. Conversely, AMH concentrations exhibited a positive correlation with TNF-α throughout the entire cohort in both univariate and multivariate analyses (p=0.007 and p=0.004, respectively), and in the multivariate model within the PCOS (p=0.004) and HPOD (p = 0.004) arms. BMI exhibited a significant positive correlation with CRP in multivariate models (p<0.001) across all studied groups, and in the PCOS arm also in the univariate model (p<0.001). In the PCOS arm, the univariate model indicated that BMI was positively correlated with TNF-α (p=0.007). FI concentrations and HOMA-IR values were significantly positively associated with CRP in multivariate models (all p-values <0.001) across all studied groups, and in the PCOS group, this association was also present in the univariate model (both p<0.001). Furthermore, FI concentrations showed a positive correlation with TNF-α in univariate models within the entire cohort and the HPOD arm (both p=0.047). In the PCOS arm, univariate models revealed that FI concentrations and HOMA-IR were positively associated with SCH (p=0.03 and p=0.001) and negatively associated with TAI (p=0.017 and p=0.022, respectively). HOMA-IR value correlated positively with SCH in multivariate models in all studied groups (all p-values< 0.008), while in PCOS additionally in univariate model (p=0.001). Total cholesterol concentration correlated positively with TAI in PCOS univariate analysis (p=0.016). FSH concentrations showed no statistically significant relationships (p>0.05) with any inflammatory markers or thyroid parameters across all studied groups and regression models analyzed.

**Table 6:** Univariate and multivariate linear regression model for selected inflammatory markers, subclinical hypothyroidism (SCH) and thyroid autoimmunity (TAI) with AMH, FSH, BMI, fasting insulin, HOMA-IR and total cholesterol as the dependent variable.

Dependent variable	Predictor	Entire cohort				PCOS				HPOD			
		Univariate linear regression		Multivariate linear regression		Univariate linear regression		Multivariate linear regression		Univariate linear regression		Multivariate linear regression	
		β	*p*	β	*p*	β	*p*	β	*p*	β	*p*	β	*p*
AMH [pmol/l]	CRP [mg/l]	-0.002	0.598	-0.007	0.096	-0.005	0.146	-0.007	0.096	0.005	0.890	-0.007	0.096
	IL-6 [pg/ml]	-0.001	0.786	0.000	0.960	-0.002	0.572	<0.001	0.960	-0.037	0.312	0.000	0.960
	IL-10 [pg/ml]	-0.002	0.011*	-0.002	0.014*	-0.001	0.155	-0.002	0.014*	0.000	0.765	-0.002	0.014*
	TNF-α [pg/ml]	0.012	0.007*	0.014	0.004*	0.007	0.066	0.014	0.004*	0.034	0.073	0.014	0.004*
	IL-1β [pg/ml]	0.017	0.674	0.006	0.881	0.003	0.934	0.006	0.881	0.054	0.801	0.006	0.881
	SCH	0.015	0.569	-0.002	0.951	-0.010	0.678	-0.002	0.951	-0.016	0.865	-0.002	0.951
	TAI	-0.015	0.601	-0.040	0.173	0.007	0.787	-0.040	0.173	0.037	0.649	-0.040	0.173
FSH [mIU/ml]	CRP [mg/l]	0.007	0.908	0.007	0.327	0.007	0.262	0.007	0.327	0.007	0.908	0.007	0.327
	IL-6 [pg/ml]	-0.029	0.650	0.004	0.513	0.006	0.394	0.004	0.513	-0.029	0.650	0.004	0.513
	IL-10 [pg/ml]	0.001	0.518	0.001	0.620	0.000	0.811	0.001	0.620	0.001	0.518	0.001	0.620
	TNF-α [pg/ml]	-0.058	0.081	0.005	0.498	0.007	0.353	0.005	0.498	-0.058	0.081	0.005	0.498
	IL-1β [pg/ml]	-0.092	0.804	-0.061	0.401	-0.039	0.560	-0.061	0.401	-0.092	0.804	-0.061	0.401
	SCH	0.100	0.537	-0.005	0.916	-0.009	0.845	-0.005	0.916	0.100	0.537	-0.005	0.916
	TAI	-0.113	0.410	-0.022	0.655	-0.010	0.829	-0.022	0.655	-0.113	0.410	-0.022	0.655
BMI [kg/m^2^]	CRP [mg/l]	0.457	0.395	0.952	<0.001*	1.020	<0.001*	0.952	<0.001*	0.457	0.395	0.952	<0.001*
	IL-6 [pg/ml]	0.461	0.386	0.070	0.610	0.221	0.181	0.070	0.610	0.461	0.386	0.070	0.610
	IL-10 [pg/ml]	-0.012	0.349	-0.009	0.677	-0.006	0.880	-0.009	0.677	-0.012	0.349	-0.009	0.677
	TNF-α [pg/ml]	0.365	0.205	0.178	0.270	0.479	0.007*	0.178	0.270	0.365	0.205	0.178	0.270
	IL-1β [pg/ml]	1.771	0.573	-0.837	0.563	1.653	0.313	-0.837	0.563	1.771	0.573	-0.837	0.563
	SCH	0.411	0.766	1.005	0.272	2.114	0.059	1.005	0.272	0.411	0.766	1.005	0.272
	TAI	2.014	0.069	-0.699	0.474	-1.310	0.274	-0.699	0.474	2.014	0.069	-0.699	0.474
Fasting insulin [uU/ml]	CRP [mg/l]	0.139	0.303	0.057	<0.001*	0.070	<0.001*	0.057	<0.001*	0.139	0.303	0.057	<0.001*
	IL-6 [pg/ml]	0.078	0.566	0.016	0.292	0.025	0.133	0.016	0.292	0.078	0.566	0.016	0.292
	IL-10 [pg/ml]	-0.003	0.384	-0.001	0.637	0.000	0.975	-0.001	0.637	-0.003	0.384	-0.001	0.637
	TNF-α [pg/ml]	0.139	0.047*	0.013	0.483	0.031	0.082	0.013	0.483	0.139	0.047*	0.013	0.483
	IL-1β [pg/ml]	-0.966	0.214	0.107	0.509	0.262	0.107	0.107	0.509	-0.966	0.214	0.107	0.509
	SCH	-0.078	0.823	0.109	0.285	0.241	0.030*	0.109	0.285	-0.078	0.823	0.109	0.285
	TAI	0.376	0.191	-0.201	0.067	-0.282	0.017*	-0.201	0.067	0.376	0.191	-0.201	0.067
HOMA-IR [n]	CRP [mg/l]	-0.016	0.885	0.076	<0.001*	0.083	<0.001*	0.076	<0.001*	-0.016	0.885	0.076	<0.001*
	IL-6 [pg/ml]	0.094	0.392	0.011	0.470	0.021	0.220	0.011	0.470	0.094	0.392	0.011	0.470
	IL-10 [pg/ml]	-0.001	0.763	-0.002	0.489	-0.001	0.786	-0.002	0.489	-0.001	0.763	-0.002	0.489
	TNF-α [pg/ml]	0.080	0.173	-0.007	0.698	0.019	0.291	-0.007	0.698	0.080	0.173	-0.007	0.698
	IL-1β [pg/ml]	-0.780	0.218	0.002	0.991	0.157	0.352	0.002	0.991	-0.780	0.218	0.002	0.991
	SCH	-0.189	0.501	0.272	0.008*	0.384	0.001*	0.272	0.008*	-0.189	0.501	0.272	0.008*
	TAI	0.243	0.306	-0.170	0.117	-0.280	0.022*	-0.170	0.117	0.243	0.306	-0.170	0.117
Total cholesterol [mmol/l]	CRP [mg/l]	-0.089	0.556	0.004	0.854	0.009	0.606	0.004	0.854	-0.089	0.556	0.004	0.854
	IL-6 [pg/ml]	-0.080	0.597	-0.004	0.825	-0.001	0.958	-0.004	0.825	-0.080	0.597	-0.004	0.825
	IL-10 [pg/ml]	-0.001	0.774	0.004	0.177	0.007	0.139	0.004	0.177	-0.001	0.774	0.004	0.177
	TNF-α [pg/ml]	0.021	0.796	0.032	0.135	0.036	0.068	0.032	0.135	0.021	0.796	0.032	0.135
	IL-1β [pg/ml]	-0.307	0.728	-0.024	0.900	0.171	0.345	-0.024	0.900	-0.307	0.728	-0.024	0.900
	SCH	-0.579	0.118	-0.120	0.325	-0.045	0.716	-0.120	0.325	-0.579	0.118	-0.120	0.325
	TAI	-0.203	0.534	0.235	0.070	0.317	0.016*	0.235	0.070	-0.203	0.534	0.235	0.070

p, univariate and multivariate linear regression model; β, regression coefficient; * statistically significant (p < 0.05); PCOS, polycystic ovary syndrome; HPOD, hypothalamic-pituitary-ovarian dysfunction; BMI, body mass index; AMH, anti-Müllerian hormone; FSH, follicle-stimulating hormone; HOMA-IR, homeostasis model assessment for insulin resistance; CRP, C-reactive protein; TNF-α, tumor necrosis factor-α.

## 4 Discussion

### 4.1 Pro-inflammatory substances in normogonadotropic anovulation

In the studied population of women with normogonadotropic anovulation, most were diagnosed with PCOS, primarily phenotype A, reflecting a broader trend in the prevalence of these disorders among reproductive-age women (4). Despite significant differences in the variables characterizing individual study subsets, no significant intergroup differences were observed in the concentrations of selected inflammatory parameters. Numerous scientific studies have examined the relationship between various pro-inflammatory factors and PCOS, with meta-analyses revealing that, among the range of investigated parameters, only the circulating CRP was significantly higher in women with PCOS compared to non-hyperandrogenic or normoovulatory controls, while the difference in IL-6 concentration remained inconsistent and the difference in TNF-α concentration was statistically non-significant (5, 6, 28). However, previous studies have not compared the concentrations of pro-inflammatory substances in PCOS and other anovulatory conditions. It is therefore possible that anovulation in PCOS may not occur with elevated serum concentrations of pro-inflammatory factors compared to other conditions of normogonadotropic anovulation.

### 4.2 Concentrations of inflammatory parameters in normogonadotropic anovulation complicated by SCH and TAI

The literature has reported a higher prevalence of SCH and TAI in reproductive-age women with PCOS compared to women without this condition (24); however, this was not a consistent finding in research employing larger sample sizes (29). The absence of intergroup differences in SCH and TAI prevalence may have resulted from the subsets not being random representative samples of the population with menstrual irregularities, as they comprised women selected for comprehensive hormonal diagnostics, potentially influencing the outcomes.

Women with SCH exhibited significantly higher median serum concentrations of CRP, TNF-α, IL-10 and IL-1β, as well as a higher CRP/SHBG ratio compared to those with normal TSH concentration, with these correlations largely consistent within the PCOS arm due to the substantial sample size, which was congruent in relation to the results of other studies (30–32). Studies have shown that levothyroxine treatment in hypothyroid patients contributes to significant decreases in IL-1 and TNF-α concentrations, supporting the role of thyroid dysfunction in promoting inflammatory states (33).

Analogous correlations were observed with respect to TAI, indicating significantly elevated TNF-α, IL-10 and IL-1β concentrations in women with circulating ATA, also apparent among those with PCOS, which was congruent in relation to the results of other studies (34). TAI involves complex inflammatory processes characterized by lymphocytic infiltration and cytokine-mediated tissue damage. The presence of TAI induces a state of chronic low-grade inflammation that may extend beyond the thyroid gland itself (34).

Conversely, no significant differences in inflammatory parameters were noted between individuals with and without SCH or TAI among women with HPOD.

The regression analysis revealed that both SCH and TAI independently influenced inflammatory parameters, particularly TNF-α concentrations, across the entire cohort and specifically within the PCOS arm. These findings suggested that thyroid dysfunction, even at subclinical levels, contributed to a pro-inflammatory state that could exacerbate the metabolic and reproductive complications associated with PCOS.

The significant elevation of the IL-1β/IL-10 ratio associated with TAI, in conjunction with the significant interaction effects between SCH and TAI observed in both the entire cohort and the PCOS arm, indicated that the interplay of SCH and TAI resulted in complex inflammatory dynamics, yielding synergistic effects on inflammatory balance that surpassed the outcomes anticipated from their individual contributions alone. This interaction indicated that the co-occurrence of these thyroid conditions might have amplified inflammatory dysregulation, particularly influencing the balance between the pro-inflammatory cytokine IL-1β and the anti-inflammatory cytokine IL-10. The IL-1β/IL-10 ratio may serve as a more sensitive indicator of immune imbalance than either cytokine alone, as it reflects the dynamic interaction between inflammatory activation and regulatory suppression (1). In contrast, the analysis revealed no significant interaction effects for TNF-α concentrations across any study groups, indicating that SCH and TAI exerted independent additive effects on this inflammatory marker. This pattern was consistent with previous research demonstrating that TAI could exert additive or synergistic effects on adverse outcomes in women with SCH (35).

The absence of significant associations in the HPOD arm may be attributed to the smaller sample or to potentially distinct underlying pathophysiological mechanisms compared to PCOS. The independent and interactive effects of SCH and TAI on inflammatory markers in PCOS underscore the clinical importance of screening for thyroid dysfunction in women with PCOS to identify those at greater risk for inflammatory complications and associated metabolic disturbances.

The significantly lower IL-6 concentration in the TAI subpopulations was contrary to findings from past studies (35), which mostly involved women diagnosed with Hashimoto’s thyroiditis, while this study included euthyroid women with incidental ATA positivity. This discrepancy may reflect different stages of TAI progression. IL-6 has complex functions beyond inflammation, including roles in regenerative processes as well as in the regulation of metabolism and both pro- and anti-inflammatory processes (36). Reduced IL-6 concentrations in the early stage of TAI provide novel insights into the pathophysiology of this condition, highlighting the need for further research into mechanisms that bypass IL-6 suppression and drive autoimmune inflammation.

### 4.3 Correlations between concentrations of inflammatory parameters and metabolic and ovarian indices in normogonadotropic anovulation complicated by SCH and TAI

The analysis revealed that among the numerous statistically significant correlations between inflammatory parameters and metabolic and ovarian indicators, those linked to carbohydrate and lipid metabolism exhibited the highest frequency and strength, further supporting the role of inflammation in the etiology of metabolic syndrome (37).

The concentration of CRP, which positively correlated with TSH, demonstrated significant associations with insulin resistance and an adverse lipid profile, particularly in SCH, with comparable correlations observed in women with PCOS. Research in women with PCOS and SCH has demonstrated significantly higher HOMA-IR values and a greater prevalence of dyslipidemia compared to the controls with normal TSH. In women with HPOD, the number of significant correlations was notably lower; however, when further analyzed by subpopulations of SCH and TAI, a significantly greater number of associations emerged, reflecting a trend similar to that seen in women with PCOS (38–40). A statistically significant positive correlation between CRP and ALT concentrations was observed throughout the entire cohort. When analyzing disease-specific subsets, this association persisted in women with PCOS and HPOD who had concurrent SCH. Notably, this correlation was not observed in women with TAI in either the PCOS or HPOD arm. These findings indicate that the relationship between systemic inflammatory activity and hepatocellular function in women with anovulation may be influenced more by thyroid functional status than by TAI. Furthermore, elevated CRP concentrations may serve as both a biomarker of systemic inflammation and a modulator of hepatic metabolic processes, particularly in the context of SCH.

For IL-6, the correlations with insulin resistance indicators were similar but less pronounced, displaying a stronger positive correlation across all indicators in SCH. The association with an unfavorable lipid profile was also evident, though weaker than that observed for CRP (32). The correlations in women with PCOS reflected those in the overall cohort; similarly, in women with HPOD, significant correlations persisted in the SCH subpopulation, mirroring the patterns seen with CRP. The positive correlations between serum concentrations of CRP and IL-6 and fT3 suggested its potential role as a biomarker of insulin resistance, as previously hypothesized (41–43).

The associations between TNF-α concentrations and insulin resistance indicators were less marked and consistently observed in SCH and TAI. The association between TNF-α concentrations and an unfavorable lipid profile remained significant for all lipid parameters, although it was weaker than that of CRP, and persisted for some indicators in SCH and TAI.

No significant correlations between IL-1β and insulin resistance were detected in the overall cohort, whereas positive correlations were noted in women with TAI (44). No significant correlations between IL-1β concentrations and lipid parameters were observed in the entire cohort, nor was there any influence from the studied thyroid dysfunctions.

As previously mentioned, the number of significant correlations between pro-inflammatory factors and ovarian parameters was substantially lower. In the SCH subpopulation of the entire cohort, a significant negative correlation between CRP concentration and estradiol was identified, whereas in TAI, a positive correlation with FSH concentration was noted.

The positive correlations between the serum concentrations of IL-1β and TNF-α and ATA concentrations supported the hypothesized role of a pro-inflammatory state in the pathogenesis of TAI (36, 45). The maintenance of ovarian function is critically dependent on the precise regulation and balance of inflammatory markers, with imbalances leading to ovarian dysfunction and impaired follicular development (2). In the context of reproductive health, significant attention was directed toward the complex relationship between inflammatory processes and ovarian reserve function (9). Research has suggested that TNF-α may act as a survival factor in granulosa cells, with the protective effects mediated through the TNF- α Receptor 2 pathway that upregulates antiapoptotic gene expression, indicating that moderate inflammatory processes may be necessary for maintaining optimal ovarian function (46). Furthermore, correlation analysis confirmed strong positive associations between body weight, BMI, and serum concentrations of CRP, IL-6, and TNF-α. These relationships were consistent across the entire cohort and within most subsets, further underscoring the role of excess adiposity in driving systemic low-grade inflammation in women with normogonadotropic anovulation.

### 4.4 Multiple regression analysis of inflammatory parameters and metabolic and ovarian indices

The analysis of associations between metabolic and ovarian parameters and the concentrations of the studied cytokines revealed significant relationships, suggesting a complex interplay of inflammatory processes in the pathophysiology of ovulatory disorders. The inverse correlation between AMH and IL-10 concentrations, coupled with the positive correlation between AMH and TNF-α concentrations, suggests that both pro-inflammatory factors and inflammatory imbalance may modulate ovarian reserve, consistent with previous observations in PCOS (46). The positive associations of BMI, FI concentration, and HOMA-IR with CRP concentrations – and, in some analyses, with TNF-α concentrations – confirm the role of chronic low-grade inflammation in the development of insulin resistance, particularly in the presence of SCH, as previously demonstrated in both population-based and PCOS-specific cohorts (30, 31). Furthermore, the positive correlation of total cholesterol concentration with TAI, and the negative associations between TAI and insulin resistance parameters in PCOS, suggest that immune-mediated mechanisms may differentially affect lipid and carbohydrate metabolism (41). The absence of significant relationships between FSH and inflammatory markers supports the notion that the regulation of FSH secretion within the hypothalamic-pituitary-ovarian axis is largely unaffected by systemic inflammatory activity (1). Previous prospective cohort analysis demonstrated that although SCH did not exert a clinically significant effect on ovarian function measures, TAI was associated with distinct correlations with gonadotropin and androgen concentrations in both PCOS and HPOD subgroups, highlighting the potential for subtle ovarian involvement in thyroid-related dysfunction (47).

The study results have demonstrated that SCH and TAI contribute to the development of a pro-inflammatory state, which may exacerbate metabolic complications associated with PCOS and potentially impact reproductive outcomes. The independent additive effects of these conditions on inflammatory markers suggest that screening for thyroid dysfunction in women with ovulatory disorders may have clinical significance for identifying those at increased risk of metabolic complications. Future studies should explore whether the treatment of SCH in this population leads to enhancements in inflammatory profiles and metabolic parameters.

### 4.5 Study strengths and limitations

The key strengths of this study include the prospective design with comprehensive hormonal, metabolic, and inflammatory assessment across well-defined subgroups stratified by both PCOS phenotypes and HPOD. The simultaneous evaluation of multiple inflammatory markers, including both pro- and anti-inflammatory cytokines, enabled a more nuanced assessment of immune imbalance beyond single biomarker approaches. Additionally, the application of multivariate regression models with interaction analysis enhanced the robustness of findings by controlling for confounders while examining independent and synergistic effects of SCH and TAI.

Several limitations should be acknowledged. The cross-sectional design precludes establishing causality between thyroid dysfunction and inflammatory parameters. The relatively small HPOD subgroup may have limited statistical power to detect significant associations. Additionally, we did not assess thyroid hormone replacement therapy effects on inflammatory markers, which represents an important area for future research.

### 4.6 Conclusions

SCH and TAI independently and synergistically promote chronic low-grade inflammation in women with normogonadotropic anovulation, particularly in PCOS, through elevated TNF-α and disruption of the IL-1β/IL-10 balance. Their strong associations with insulin resistance underscore the need for thyroid screening in metabolic evaluation, while the inverse correlation of AMH with IL-10 and its positive link with TNF-α suggest a direct role of inflammation in modulating ovarian reserve. The absence of similar findings in HPOD indicates distinct pathophysiological mechanisms requiring further study.

## Data Availability

The datasets presented in this study can be found in online repositories. The names of the repository/repositories and accession number(s) can be found in the article/**Supplementary Material**.
